# Comparison of T2N0M0 and T3aN0M0 in Predicting the Prognosis of Patients With Renal Cell Carcinoma

**DOI:** 10.3389/fonc.2020.564631

**Published:** 2020-09-23

**Authors:** Xiaobo He, Xiaopeng Mao, Jibin Li, Shengjie Guo

**Affiliations:** ^1^State Key Laboratory of Oncology in South China, Department of Urology, Collaborative Innovation Center for Cancer Medicine, Sun Yat-sen University Cancer Center, Guangzhou, China; ^2^Department of Urology Surgery, The First Affiliated Hospital, Sun Yat-sen University, Guangzhou, China; ^3^State Key Laboratory of Oncology in South China, Department of Clinical Research, Collaborative Innovation Center for Cancer Medicine, Sun Yat-sen University Cancer Center, Guangzhou, China

**Keywords:** renal carcinoma, radical surgery, tumor size, prognosis, TNM classification

## Abstract

**Background:** To investigate the prognostic role of tumor size in patients with pathological T2N0M0 and T3aN0M0 renal cell carcinoma (RCC) treated by radical surgery.

**Methods:** A total of 3,662 cases were retrospectively analyzed from the Surveillance, Epidemiology and End Results (SEER) from 2010 to 2012. Overall survival (OS) and cancer-specific survival (CSS) data were obtained. The log-rank test was used to compare survival distributions and Cox proportional hazards model was used for univariate and multivariate analyses, respectively.

**Results:** In the low-risk T3aN0M0 (perinephric fatty infiltration or sinus fatty infiltration only) group, patients with tumor size ≤ 7 cm were associated with a better OS (*P* = 0.009) and CSS (*P* < 0.001) than those with tumor size >7 cm. However, there was no difference in OS (*P* = 0.129) and CSS (*P* = 0.539) between T2bN0M0 patients and low-risk T3aN0M0 patients with tumor size ≤ 7 cm. A new T classification grouping patients with both T2bN0M0 and T3aN0M0 with tumor diameter ≤ 7 cm into the same staging category (pT2aN0M0, pT2bN0M0+low-risk pT3aN0M0 [tumor diameter ≤ 7cm], low-risk pT3aN0M0 [tumor diameter >7 cm], high-risk pT3aN0M0) was proposed and it was found as an independent predictive variable for OS and CSS.

**Conclusions:** Findings from the present study suggest that the reclassification of pT2N0M0 and pT3aN0M0 RCC can lead to better prediction of OS and CSS.

## Introduction

Renal cell carcinoma (RCC) is the most frequently diagnosed cancer of kidney. It is estimated that over 73,750 new cases will be diagnosed in 2020, of which approximately 14,830 will die of RCC (1). According to the pathological classification, RCC consists of clear cell RCC, papillary RCC, chromophobe RCC, unclassified RCC and other subtypes (2, 3). Nowadays, the most important prognostic indicator of RCC is the tumor, lymph node and metastasis (TNM) classification, providing patients with important prognostic and therapeutic information (4–6). In recent decades, the gold standard system has been continuously revised to improve its prognostic accuracy and predictive ability (7). According to the 7th edition of AJCC TNM staging, the pathological stage T2 RCC is only classified on the basis of tumor size, while T3a is defined based on anatomical tumor expansion, including fatty infiltration or venous invasion, irrespective of tumor size (8). Therefore, considering the tumor size for classification, this may indicate that T3a can be further classified and modified.

Several studies have investigated the prognostic significance of tumor size in patients with pT3a RCC (9–16) and surgery remains the most important form of treatment for resectable cases, despite the implementation of novel therapeutics (17). In addition, we have reported the outcomes of 1,869 patients receiving radical nephrectomy from the Surveillance, Epidemiology and End Results (SEER) database, and we have demonstrated that different invasion locations can help distinguish T3aN0M0 clear cell RCC patients with increased risk of cancer-related mortality (18). In addition, Laguna has suggested that the pT3a category is still heterogeneous despite changes in the 7th TNM classification. The prognosis of “low-risk pT3a disease” (perinephric fatty infiltration [PFI] or sinus fatty infiltration [SFI] only) and “high-risk pT2 disease” may need to be further compared, not only to redefine pT3a disease, but also to clarify possible overlaps with pT2b categories and imply that the tumor size is consistently a strong prognostic factor for RCC (19). Uniquely, the TNM system subdivides pT2a and pT2b RCCs according to tumor size alone, and our previous study has shown that pT3a patients with different invasion patterns have different prognoses, indicating that patients with pT2 and pT3a tumors constitute a very heterogeneous population, at least with regard to tumor size or invasion pattern. Therefore, there are questionable differences in the prognostic significance of pT2N0M0 and pT3aN0M0 with only PFI or SFI in RCC patients. To examine such hypothesis, we conducted the difference in prognosis between pT2N0M0 and pT3aN0M0 RCC patients who underwent radical surgery and to compute a model for stratifying their outcome based on the SEER dataset.

## Methods

### Patients and Study Design

Unidentified patient data were obtained from the SEER program, which were composed of 18 population-based registries, accounting for approximately 28% of the US population (https://seer.cancer.gov/, accession number: 14558-Nov2018). SEER program is populated with high-quality population-based data from national cancer registries. The crucial status is updated once a year, and quality control checks are regularly performed.

All patients were diagnosed with RCC according to International Classification of Diseases-O-3 (ICD-O-3) codes C64.9 between January 2010 and December 2012 in the SEER. The following variables were collected and coded: age at diagnosis, race recode, sex, year of diagnosis, AYA site recode, ICCC site recode ICD-O-3/WHO 2008, primary site, histological type ICD-O-3, grade, laterality, American Joint Commission on Cancer (AJCC) 7th edition TNM system, surgery of primary site, CS tumor size, CS extension, CS site-specific factor 1, SEER cause-specific death classification, survival time and vital status. Subsequently, 3,662 cases were included from the dataset according to our inclusion and exclusion criteria (Supplementary Figure 1). Based on previous studies (16, 18), we defined the low-risk T3aN0M0 as one pattern of extrarenal extension (PFI or SFI) and the high-risk T3aN0M0 as multiple pattern of extrarenal extension (PFI+SFI, PFI+ renal vein infiltration [RVI], SFI+RVI, PFI+SFI+RVI). The clinical data used in this study were obtained from the SEER database, a public research resource that does not require patient consent and ethical consent.

### Outcomes

Cancer-specific survival (CSS) and Overall survival (OS), which were coded by SEER, were included in this study.

### Statistical Analysis

Baseline characteristics were analyzed using descriptive statistics. To define an appropriate cut-off value for segmenting patients with T3aN0M0 RCC based on tumor size, Martingale residuals (20) were computed from the Cox proportional hazards model, and the residuals were subsequently plotted against the tumor size to identify the cut-off value, as mentioned in the previous study (7). Kaplan-Meier curves were generated to assess outcomes, and differences between groups were compared using log-rank analysis. Cox regression analysis was used for factors with statistical significance in univariate and multivariate analysis. All analyses were performed using SPSS software ver. 25.0 (IBM, Armonk, NY, USA) and EmpowerStats software (www.empowerstats.com, X&Y solutions, Inc. Boston MA). All tests were two-sided and a *P* < 0.05 was considered as statistically significant.

## Results

### Baseline Characteristics of Patients

A total of 3,662 patients were included in the study, and Table 1 summarizes their baseline characteristics. Of these patients, 2,439 patients (66.60%) were males, and 1,223 (33.40%) were females. Among patients with a given stage, 1,450 patients (39.60%) were diagnosed with pT2aN0M0, 624 patients (17.04%) were diagnosed with pT2bN0M0, 989 patients (27.00%) were diagnosed with low-risk pT3aN0M0, and 599 patients (16.36%) were diagnosed with high-risk pT3aN0M0. Moreover, 1,882 patients (51.39%) had tumors on the left side, and 1,780 (48.61%) had tumors on the right side. The median duration of follow-up was 64.00 months. 993 (27.10%) of 3,662 patients died during the follow-up period.

**Table 1:** Clinical and tumor characteristics of the 3,662 analyzed enrollees.

**Variables**	**Total (*N* = 3,662)**	**T2aN0M0 (*N* = 1,450)**	**T2bN0M0 (*N* = 624)**	**T3aN0M0 (*****N*** **=** **1,588)**	
				**Low-risk T3aN0M0**	**High-risk T3aN0M0**
				**(*N* = 989)**	**(*N* = 599)**
Age at diagnosis (years) (median [min-max])	63 (18–98)	61 (18–94)	59 (21–90)	65 (24–98)	66 (30–92)
Tumor size (mm) (Mean ± SD)	88.74 ± 36.72	83.49 ± 8.63	132.68 ± 47.88	72.27 ± 34.87	82.85 ± 33.36
**Year of diagnosis**					
2010	1,249 (34.11%)	480 (33.10%)	199 (31.89%)	369 (37.31%)	201 (33.56%)
2011	1,189 (32.47%)	465 (32.07%)	219 (35.10%)	311 (31.45%)	194 (32.39%)
2012	1,224 (33.42%)	505 (34.83%)	206 (33.01%)	309 (31.24%)	204 (34.06%)
**Race**					
White	3,054 (83.40%)	1,183 (81.59%)	493 (79.01%)	848 (85.74%)	530 (88.48%)
Black	390 (10.65%)	170 (11.72%)	94 (15.06%)	91 (9.20%)	35 (5.84%)
Other (American Indian/AK Native, Asian/Pacific Islander)	204 (5.57%)	90 (6.21%)	37 (5.93%)	46 (4.65%)	31 (5.18%)
Unknown	14 (0.38%)	7 (0.48%)	0 (0.00%)	4 (0.40%)	3 (0.50%)
**Sex**					
Male	2,439 (66.60%)	905 (62.41%)	427 (68.43%)	688 (69.57%)	419 (69.95%)
Female	1,223 (33.40%)	545 (37.59%)	197 (31.57%)	301 (30.43%)	180 (30.05%)
**Grade**					
Well-differentiated; Grade I	227 (6.20%)	127 (8.76%)	34 (5.45%)	50 (5.06%)	16 (2.67%)
Moderately differentiated; Grade II	1,597 (43.61%)	728 (50.21%)	296 (47.44%)	397 (40.14%)	176 (29.38%)
Poorly differentiated; Grade III	1,420 (38.78%)	492 (33.93%)	246 (39.42%)	405 (40.95%)	277 (46.24%)
Undifferentiated; anaplastic; Grade IV	418 (11.41%)	103 (7.10%)	48 (7.69%)	137 (13.85%)	130 (21.70%)
**Laterality**					
Left - origin of primary	1,882 (51.39%)	741 (51.10%)	308 (49.36%)	513 (51.87%)	320 (53.42%)
Right - origin of primary	1,780 (48.61%)	709 (48.90%)	316 (50.64%)	476 (48.13%)	279 (46.58%)
**Histological subtype**					
Clear cell RCC	2,429 (66.33%)	954 (65.79%)	321 (51.44%)	674 (68.15%)	480 (80.13%)
Papillary RCC	410 (11.20%)	172 (11.86%)	120 (19.23%)	97 (9.81%)	21 (3.51%)
Chromophobe RCC	272 (7.43%)	112 (7.72%)	87 (13.94%)	63 (6.37%)	10 (1.67%)
Collecting duct RCC	10 (0.27%)	2 (0.14%)	0 (0.00%)	5 (0.51%)	3 (0.50%)
RCC, unclassified	440 (12.02%)	181 (12.48%)	89 (14.26%)	105 (10.62%)	65 (10.85%)
Translocation RCC	21 (0.57%)	6 (0.41%)	1 (0.16%)	12 (1.21%)	2 (0.33%)
Sarcomatoid RCC	43 (1.17%)	8 (0.55%)	1 (0.16%)	19 (1.92%)	15 (2.50%)
Others	37 (1.01%)	15 (1.03%)	5 (0.80%)	14 (1.42%)	3 (0.50%)

### Survival Analyses for T2N0M0 and T3aN0M0 Patients

**Figure 1:**
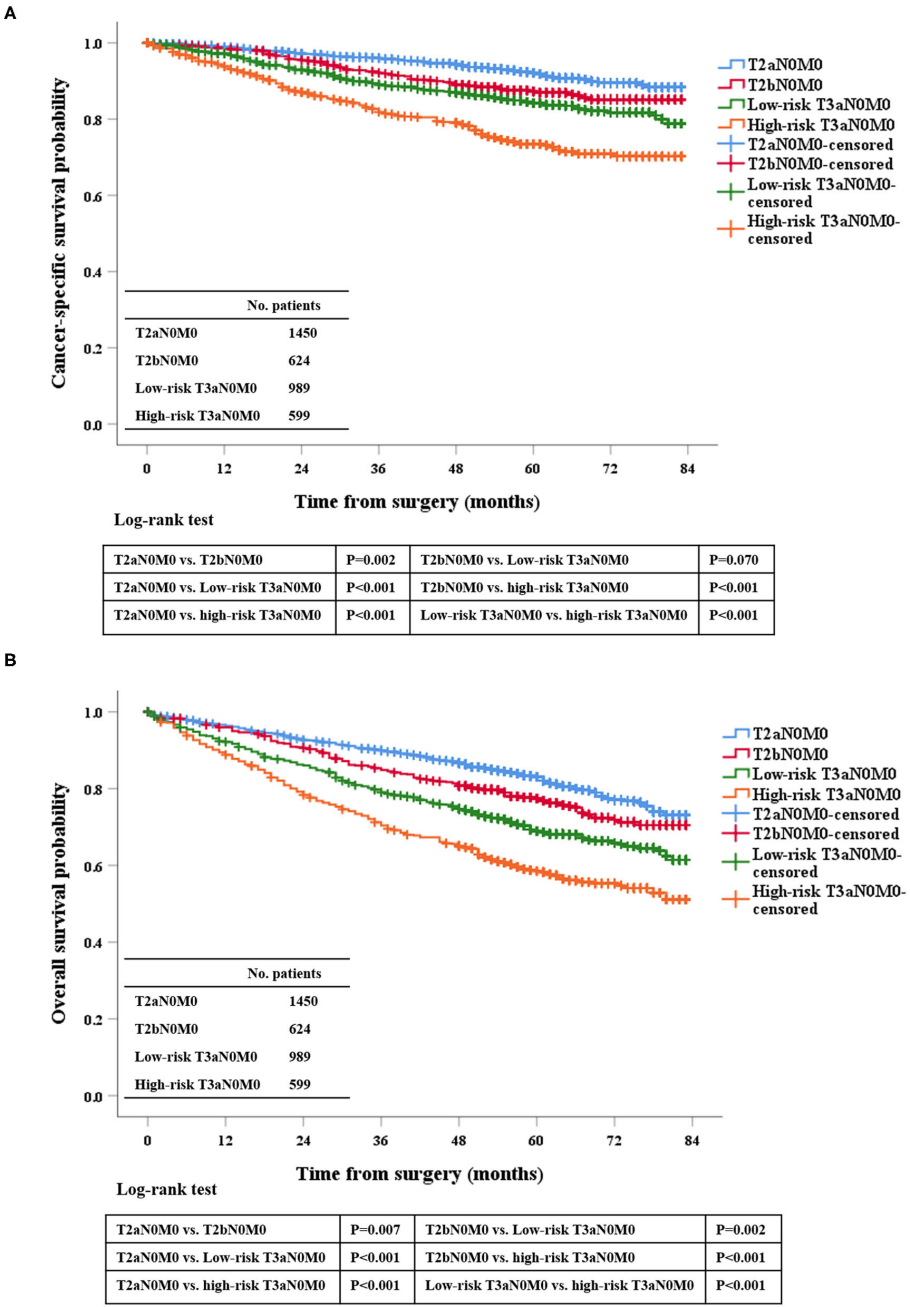
Kaplan-Meier survival estimates for patients with pT2aN0M0, pT2bN0M0, low-risk pT3aN0M0, and high-risk pT3aN0M0 RCC who underwent radical surgery for CSS **(A)** and OS **(B)**.

We analyzed the prognosis of the related patients according to the 7th TNM system. First, OS and CSS among pT2aN0M0, pT2bN0M0, low-risk pT3aN0M0, and high-risk T3aN0M0 groups were statistically significant (each *P* < 0.050; Figure 1), except for pT2bN0M0 vs. low-risk pT3aN0M0 in CSS (*P* = 0.070; Figure 1). This finding showed that those current T-stage classifications could grade patients for prognosis. Second, for low-risk pT3aN0M0 RCC patients, the OS and CSS of pT3aN0M0 patients with PFI only were similar to those of patients with SFI only (CSS, *P* = 0.509; OS, *P* = 0.519; Supplementary Figure 2). However, tumor size was not considered in this classification.

### Identification of a Cut-Off Value of Tumor Size for the Low-Risk T3aN0M0 Patients

**Figure 2:**
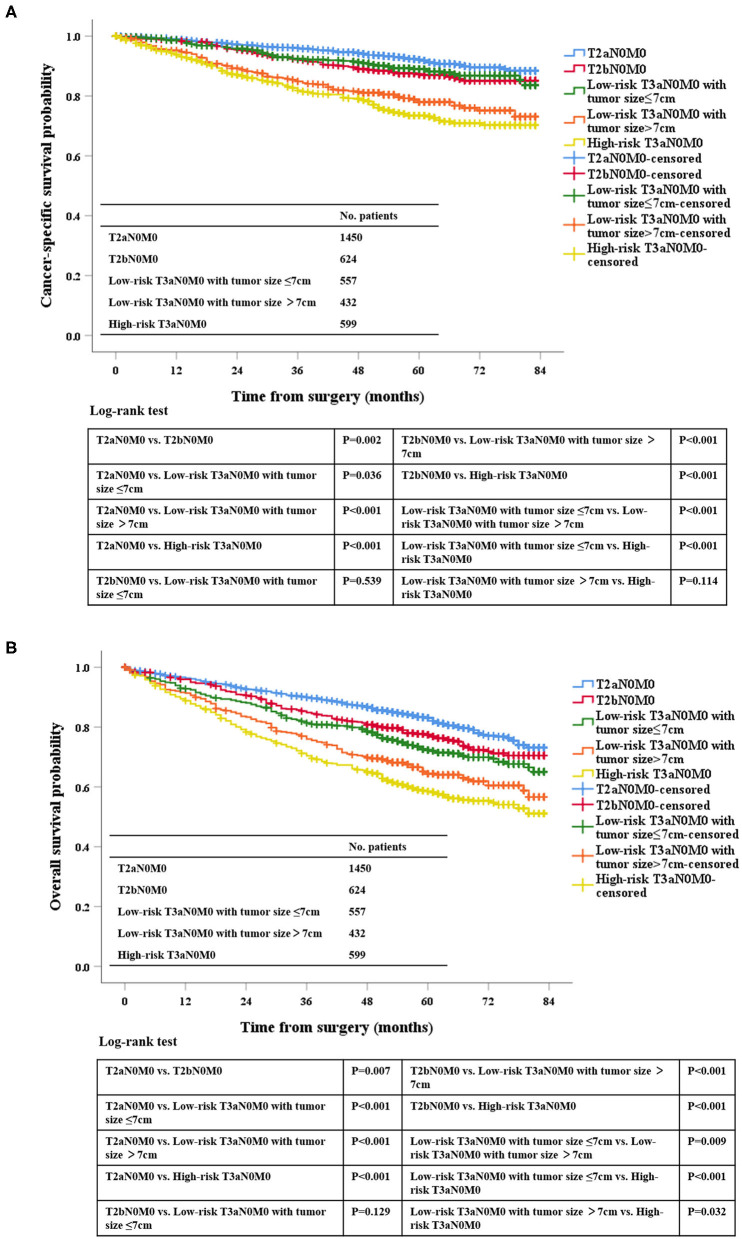
Kaplan-Meier analysis of the survival of low-risk pT3aN0M0 RCC patients after radical surgery stratified by tumor size with a cut-off of 7 cm in all cohorts for CSS **(A)** and OS **(B)**.

Univariate analysis was performed in low-risk pT3aN0M0 RCC patients, and the tumor size was significantly correlated with OS (HR: 1.005, 95%CI: 1.002–1.007, *P* = 0.003) and CSS (HR: 1.011, 95%CI: 1.007–1.015, *P* < 0.001), indicating that for every 1 mm increase in tumor size lead to a higher risk of death of OS and CSS. In addition, Martingale residuals revealed that an appropriate cut-off value was 7 cm (Supplementary Figure 3). According to the cut-of value to subdivide this group, our data demonstrated that the OS and CSS were significantly improved for the low-risk pT3aN0M0 patients with tumors ≤ 7 cm compared with the low-risk pT3aN0M0 patients with tumors > 7 cm by the Kaplan-Meier curves and log-rank test (OS, *P* = 0.009; CSS, *P* < 0.001; Figure 2). In addition, in order to distinguish whether there was a different prognosis for the patients with T2bN0M0 and Low-risk T3aN0M0 (tumor size ≤ 7 cm) between ccRCC and non-ccRCC subgroups, we further analyzed the different staging subgroups [(1) T2bN0M0, (2) Low-risk T3aN0M0 with tumor size ≤ 7 cm, (3) Low-risk T3aN0M0 with tumor size >7 cm, and (4) High-risk T3aN0M0] in the two different pathological subgroups. Based on the results of Kaplan-Meier curves, for OS, there was no significance between the patients with T2bN0M0 and Low-risk T3aN0M0 (tumor size ≤ 7 cm) in the ccRCC (*P* = 0.649) and non-clear cell RCC (P=0.126) subgroup (Supplementary Figures 4A,B). For CSS, we found that the same result (ccRCC subgroup, *P* = 0.086. non-clear cell RCC subgoup, *P* = 0.374) (Supplementary Figures 4C,D). After comprehensively analyzing above-mentioned results, we believed that the cut-off value should be 7 cm in tumor size for low-risk T3a patients.

### Risk Factors for Survival

**Figure 3:**
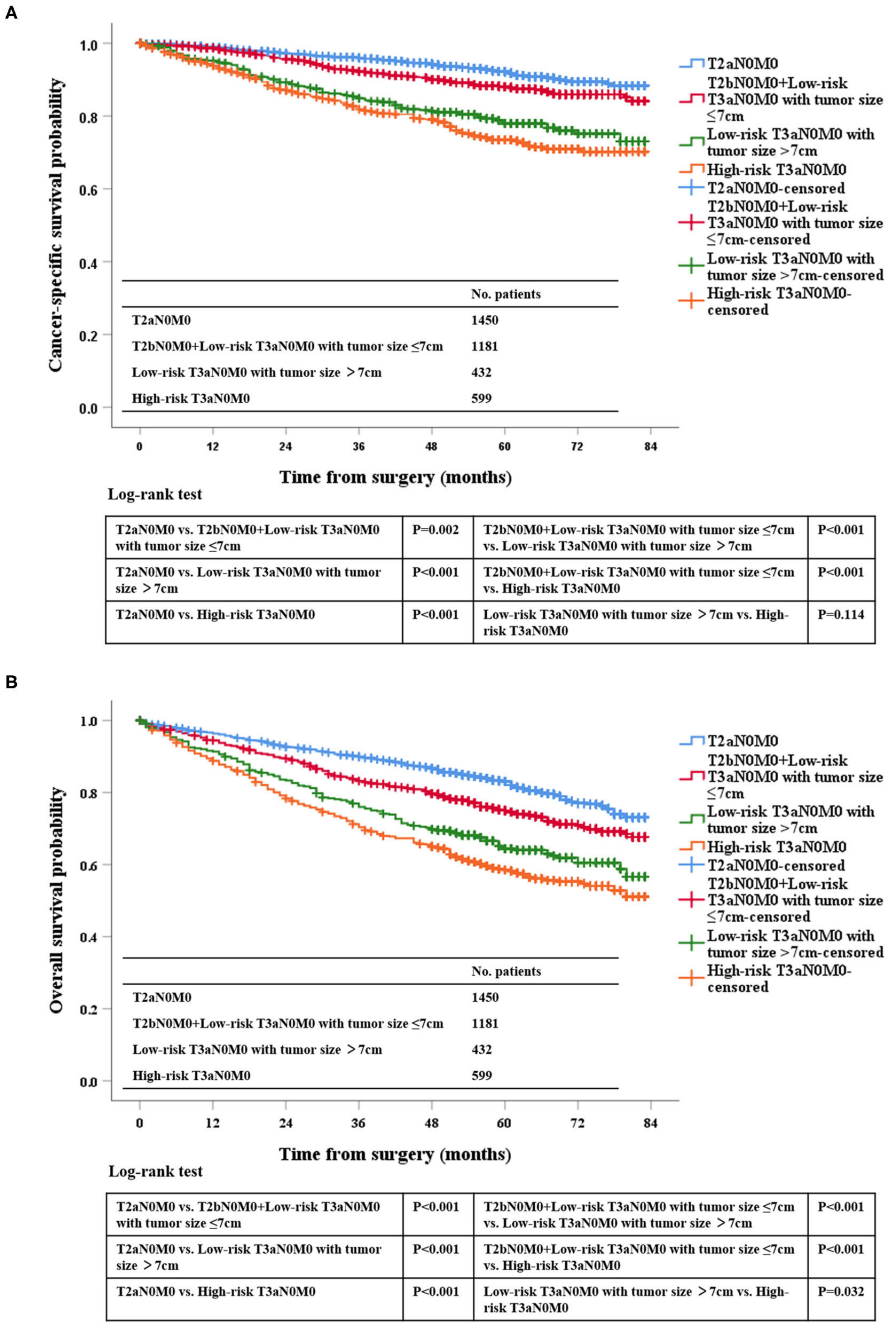
Kaplan-Meier survival estimates for pT2aN0M0, pT2bN0M0 and low-risk pT3aN0M0 RCC patients with tumor size ≤7 cm, low-risk pT3aN0M0 patients with tumor size of >7 cm, and high-risk pT3aN0M0 patients who underwent radical surgery for CSS **(A)** and OS **(B)**.

According to the Kaplan-Meier plot and log-rank test, we found that there was no difference in terms of OS and CSS between pT2bN0M0 patients and low-risk pT3aN0M0 patients with tumor size ≤ 7 cm (OS, *P* = 0.129; CSS, *P* = 0.539; Figure 2). Afterwards, we combined pT2bN0M0 and low-risk pT3aN0M0 patients with tumor size ≤ 7 cm into one group, our results showed that low-risk pT3aN0M0 patients with tumor size >7 cm experienced significantly worse OS and CSS compared with the remaining two groups (pT2aN0M0, or pT2bN0M0+low-risk pT3aN0M0 with tumor size ≤ 7 cm) (OS, *P* < 0.001; CSS, *P* < 0.001; Figure 3). However, For CSS, there was no statistical significance between the high-risk pT3aN0M0 and the low-risk pT3aN0M0 patients with tumor size >7 cm (*P* = 0.122; Figure 3). Data regarding age, sex, grade, histological subtype, laterality, and new T classification (pT2a, pT2b+low-risk pT3a with tumor size ≤ 7 cm, low-risk pT3a with tumor size >7 cm, high-risk pT3a) were included in univariate and multivariate Cox regression analyses. We found that the significant prognostic factors for OS were age, sex, grade, histological subtype, and new classification. Regarding CSS, the significant prognostic factors included age, grade, histological subtype, and new T classification (Table 2). Furthermore, the multivariate analysis identified that the new T classification was a prognostic indicator for OS (pT2b+low-risk pT3a with tumor size ≤ 7 cm vs. pT2a, HR = 1.28, 95%CI: 1.09–1.51, *P* = 0.003; low-risk pT3a with tumor size >7 cm vs. pT2a, HR = 1.61, 95% CI: 1.31–1.97, *P* < 0.001; high-risk pT3a vs. pT2a, HR = 1.74, 95% CI: 1.45–2.07, *P* < 0.001) and CSS (pT2b+low-risk pT3a with tumor size ≤ 7 cm vs. pT2a, HR = 1.43, 95% CI: 1.11–1.83, *P* = 0.005; low-risk pT3a with tumor size >7 cm vs. pT2a, HR = 2.10, 95% CI: 1.58–2.79, *P* < 0.001; high-risk pT3a vs. pT2a, HR = 2.43, 95% CI: 1.89–3.14, *P* < 0.001) (Table 3).

**Table 2:** Univariate analyses for overall survival (OS) and cancer-specific survival (CSS).

**Variable**	**Univariate analysis for OS**		**Univariate analysis for CSS**	
	**HR (95%CI)**	***P*-value**	**HR (95%CI)**	***P*-value**
Age at diagnosis	1.04 (1.04, 1.05)	<0.001	1.02 (1.01, 1.02)	<0.001
**Sex**
Male	1.00 (ref.)		1.00 (ref.)	
Female	0.82 (0.71, 0.94)	0.005	0.87 (0.72, 1.05)	0.166
**Grade**
Well-differentiated; Grade I	1.00 (ref.)		1.00 (ref.)	
Moderately differentiated; Grade II	1.46 (1.01, 2.10)	0.041	1.28 (0.73, 2.23)	0.376
Poorly differentiated; Grade III	2.42 (1.69, 3.46)	<0.001	2.82 (1.64, 4.85)	<0.001
Undifferentiated; anaplastic; Grade IV	4.71 (3.24, 6.83)	<0.001	6.53 (3.75, 11.34)	<0.001
**Histological subtype**
Clear cell RCC	1.00 (ref.)		1.00 (ref.)	
Papillary RCC	1.00 (0.82, 1.23)	0.949	0.59 (0.41, 0.84)	0.004
Chromophobe RCC	0.33 (0.23, 0.49)	<0.001	0.18 (0.09, 0.37)	<0.001
Collecting duct RCC	4.91 (2.33, 10.35)	<0.001	5.71 (2.13, 15.32)	0.001
RCC, unclassified	1.07 (0.89, 1.30)	0.444	1.08 (0.83,1.41)	0.551
Translocation RCC	5.10 (3.10, 8.38)	<0.001	3.15 (1.30, 7.64)	0.011
Sarcomatoid RCC	3.18 (2.12, 4.79)	<0.001	4.75 (2.95, 7.63)	<0.001
Others	1.28 (0.72, 2.27)	0.392	1.46 (0.69, 3.09)	0.317
**Laterality**
Left - origin of primary	1.00(ref.)		1.00(ref.)	
Right - origin of primary	0.91 (0.81, 1.04)	0.180	0.99 (0.83, 1.18)	0.934
**New T classification**
pT2a	1.00 (ref.)		1.00 (ref.)	
pT2b + low-risk pT3a (tumor diameter ≤ 7 cm)	1.42 (1.21, 1.67)	<0.001	1.48 (1.15, 1.90)	0.002
Low-risk pT3a (tumor diameter >7 cm)	2.09 (1.72, 2.55)	<0.001	2.85 (2.16, 3.76)	<0.001
High-risk pT3a	2.62 (2.20, 3.10)	<0.001	3.55 (2.78, 4.54)	<0.001

**Table 3:** Multivariate analyses for overall survival (OS) and cancer-specific survival (CSS).

**Variable**	**Multivariate analysis for OS**		**Multivariate analysis for CSS**	
	**HR (95%CI)**	***P*-value**	**HR (95%CI)**	***P*-value**
Age at diagnosis	1.04 (1.03, 1.05)	<0.001	1.01 (1.00, 1.02)	<0.001
**Sex**
Male	1.00 (ref.)		1.00 (ref.)	
Female	0.84 (0.73, 0.96)	0.015	0.95 (0.78, 1.15)	0.604
**Grade**
Well-differentiated; Grade I	1.00 (ref.)		1.00 (ref.)	
Moderately differentiated; Grade II	1.39 (0.96, 2.00)	0.075	1.23 (0.71, 2.15)	0.451
Poorly differentiated; Grade III	2.08 (1.45, 2.99)	<0.001	2.41 (1.40, 4.15)	0.001
Undifferentiated; anaplastic; Grade IV	3.71 (2.54, 5.43)	<0.001	4.69 (2.67, 8.23)	<0.001
**Histological subtype**
Clear cell RCC	1.00 (ref.)		1.00 (ref.)	
Papillary RCC	1.07 (0.87, 1.31)	0.502	0.70 (0.49, 1.01)	0.057
Chromophobe RCC	0.40 (0.27, 0.59)	<0.001	0.21 (0.10, 0.44)	<0.001
Collecting duct RCC	5.24 (2.48, 11.08)	<0.001	6.59 (2.45, 17.75)	<0.001
RCC, unclassified	1.09 (0.90, 1.32)	0.361	1.09 (0.83, 1.42)	0.508
Translocation RCC	2.60 (1.57, 4.31)	<0.001	1.96 (0.80, 4.79)	0.137
Sarcomatoid RCC	1.58 (1.03, 2.42)	0.036	1.95 (1.18, 3.24)	0.009
Others	1.65 (0.93, 2.93)	0.085	1.93 (0.91, 4.09)	0.086
**Laterality**
Left - origin of primary	1.00 (ref.)		1.00 (ref.)	
Right - origin of primary	0.94 (0.83, 1.07)	0.394	1.01 (0.85, 1.21)	0.852
**New T classification**
pT2a	1.00(ref.)		1.00(ref.)	
pT2b+low-risk pT3a (tumor diameter ≤ 7 cm)	1.28 (1.09, 1.51)	0.003	1.43 (1.11, 1.83)	0.005
Low-risk pT3a (tumor diameter >7 cm)	1.61 (1.31, 1.97)	<0.001	2.10 (1.58, 2.79)	<0.001
High-risk pT3aN0M0	1.74 (1.45, 2.07)	<0.001	2.43 (1.89, 3.14)	<0.001

## Discussion

Four major conclusions were drawn based on our current data regarding prognostic discrimination for RCC patients receiving radical surgery in pT2a - pT3a N0M0 group. First, our findings also validated that the prognosis of pT3aN0M0 RCC patients with PFI only was similar to that of patients with SFI only, which was consistent with our previous result and other related studies (16, 21, 22). Second, in the low-risk pT3aN0M0 RCC group after radical resection, the tumor size remained an independent prognostic signature, and a cut-off value of 7 cm provided the best possible prognostic discrimination. Moreover, pT3aN0M0 RCC patients with tumor size >7 cm exhibited a higher risk profile compared with the patients with tumor size ≤ 7 cm. The proposed cut-off value of 7 cm for the low-risk pT3aN0M0 RCC patients receiving radical surgery could avoid adding unnecessary complexity to the TNM system as it has been widely used for RCC TNM staging system. Third, the prognosis of pT2bN0M0 RCC patients receiving radical resection was very similar to that of low-risk pT3aN0M0 patients with tumor size ≤ 7 cm, which might be merged into one staging category. Fourth, our results showed significant distinctions among the patients with pT2 and pT3a tumors regarding OS and CSS according to new T classification method, namely, pT2a vs. pT2b+low-risk pT3a with tumor size ≤ 7 cm vs. low-risk pT3a with tumor size >7 cm vs. high-risk pT3a. Furthermore, the multivariate analysis identified that the new T classification was an independent prognostic indicator for OS and CSS. Our data were consistent with several previous findings (10, 23), in which the authors have also determined 7 cm as the best prognostic cut-off in the whole T3a patients. However, what our actual expectations for RCC staging are remains the first question to answer before further revising the pT staging system, which may be the best prognostic judgment. It is reasonable to redefine the pT3a population by combining tumor size and invasion site.

According to the 7 edition of TNM staging manual, classification of pT2 RCC depends solely on tumor size, whereas pT3a RCC is defined based on anatomic tumor expansion, regardless of tumor size. Although the TNM staging has now been updated to the eighth edition (24), the staging for pT2 and pT3a remains unchanged. However, it is well-known that tumor size is an important prognostic factor for patients with RCC. In keeping with other relevant studies, our data suggested that different pattern of extrarenal extension divided related patients into low-risk and high risk pT3a and a maximum tumor size of 7 cm represented the optimal cut-off for prognostic discrimination of patients with low-risk pT3a RCC (9, 10, 12, 14, 23). Lam et al. (10) and Brookman-May et al. (23) have analyzed T3a RCC patients and identified an ideal tumor size cut-off of 7 cm, although some patients have lymph node metastases or distant metastases. Chen et al. (14) have demonstrated that the tumor size significantly affects the survival outcomes of pT3aN0M0 RCC patients undergoing radical nephrectomy, and a cut-off size of 7 cm can help enhance the prognostic discrimination. These findings indicate the prognostic differentiation of low-risk pT3a tumors on basis of tumor size and the prognostic similarity between them and pT2 tumors. On the other hand, different pathological types of RCC also play an important role in the prognosis of patients and may have different propensity to develop renal sinus fat and renal vein invasion (25). To substantiate the proposal to combine pT2b and low-risk pT3a (tumor size ≤ 7 cm), we further analyzed whether there was a prognostic difference between pT2b and low-risk pT3a (tumor size ≤ 7 cm) for the subgroups of ccRCC and non-clear cell RCC. We found that there was no significance between the patients with T2b and low-risk T3a (tumor size ≤ 7 cm) in the ccRCC and non-clear cell RCC subgroups for OS and CSS. Furthermore, to the best of our knowledge, no study has reported a clear size threshold in total resection of low-risk T3a patients with RCC. An important implication of this study was that patients with low-risk pT3aN0M0 RCC could be further divided into two groups, and more attention should be paid to the subgroup with a tumor size <7 cm. These tumors were associated with a significantly increased risk of mortality, suggesting that closer surveillance might be warranted in such patients. Given such information, postoperative treatments for populations with different risks should be optimized, such as new adjuvant drugs. Additional research is required to discover other tumor characteristics, which are identified by using molecular or specialized imaging techniques to assist in risk stratification.

The present study has several limitations. First, all data were obtained from the SEER database. As there was no information on basic laboratory parameters and postoperative treatment in the database, the power to identify potential associations was limited. Second, we only selected patients who underwent total resection in order to ensure the accuracy of the size, which also reduced the sample size. Third, there was a lack of definite information on cases of individual RVI-only in the SEER database. Despite these limitations, as far as we know, this was the first study that compared the prognosis between pT2N0M0 and pT3aN0M0 patients with RCC who underwent radical surgery.

## Conclusions

Our results indicated that tumor size has a significant impact on the survival outcomes of patients with low-risk pT3aN0M0 RCC undergoing radical surgery. The prognosis of low-risk pT3aN0M0 RCC patients with tumor size ≤ 7 cm might be similar to that of patients with pT2bN0M0 tumors, while low-risk pT3aN0M0 patients with tumor size > 7 cm may have a worse prognosis than patients with pT2N0M0 in RCC. Above all, For RCC patients who underwent radical surgery, we proposed a new T classification (pT2a, pT2b+low-risk pT3a [tumor diameter ≤ 7 cm], low-risk pT3a [tumor diameter >7 cm], high-risk pT3a).

## Data Availability Statement

Publicly available datasets were analyzed in this study. This data can be found here: Surveillance, Epidemiology, and End Results (SEER) database (https://seer.cancer.gov/).

## Author Contributions

All authors listed have made a substantial, direct and intellectual contribution to the work, and approved it for publication.

## Conflict of Interest

The authors declare that the research was conducted in the absence of any commercial or financial relationships that could be construed as a potential conflict of interest.

## Supplementary Material

Supplementary Figure 1 Flow diagram for the patient selection from SEER. The encoding of the database is as follows. The renal cancer coded as primary site (C64.9), AYA site recode (8.5.1 carcinoma of kidney), and ICCC site recode ICD-O-3/WHO 2008 [IV (b) renal cancer]. Radical surgery coded as surgery of primary site (40 or 50). The invasion site of PFI coded as CS site-specific factor 1 (10) with CS extension (450 or 460). SFI coded as CS site-specific factor 1 (20) with CS extension (450 or 460). PFI+SFI coded as CS site-specific factor 1 (30) with CS extension (450 or 460). PFI+RVI coded as CS site-specific factor 1 (10) with CS extension (601). SFI+RVI coded as CS site-specific factor 1 (20) with CS extension (601). PFI+SFI+RVI coded as CS site-specific factor 1 (30) with CS extension (601).

Supplementary Figure 2 Kaplan-Meier survival estimates for low-risk pT3aN0M0 RCC patients undergoing radical surgery with PFI only and SFI only for CSS **(A)** and OS **(B)**.

Supplementary Figure 3 Scatterplot of tumor size vs. Martingale residuals for 989 low-risk T3aN0M0 RCC patients undergoing radical surgery based on the CSS **(A)** and OS **(B)**. The Martingale residual is the difference between the observed event and the expected numbers of events for a given patient, which indicates the expected risk of death for these patients. Compared with the expected risk based on Cox regression analysis, patients above the level line have a higher risk of death, while those below the level line have a lower risk of death. The figure shows that it is appropriate to distinguish these patients by 7 cm.

Supplementary Figure 4 Kaplan-Meier survival estimates for different staging patients undergoing radical surgery in the ccRCC and non-clear cell ccRCC subgroups for OS **(A,B)** and CSS **(C,D)**.
