# Supplementary figures and images for: Comparison of T2N0M0 and T3aN0M0 in Predicting the Prognosis of Patients With Renal Cell Carcinoma
Source: Front Oncol. 2020 Sep 23;10:564631. doi: 10.3389/fonc.2020.564631 (PMC7539120; doi:10.3389/fonc.2020.564631)

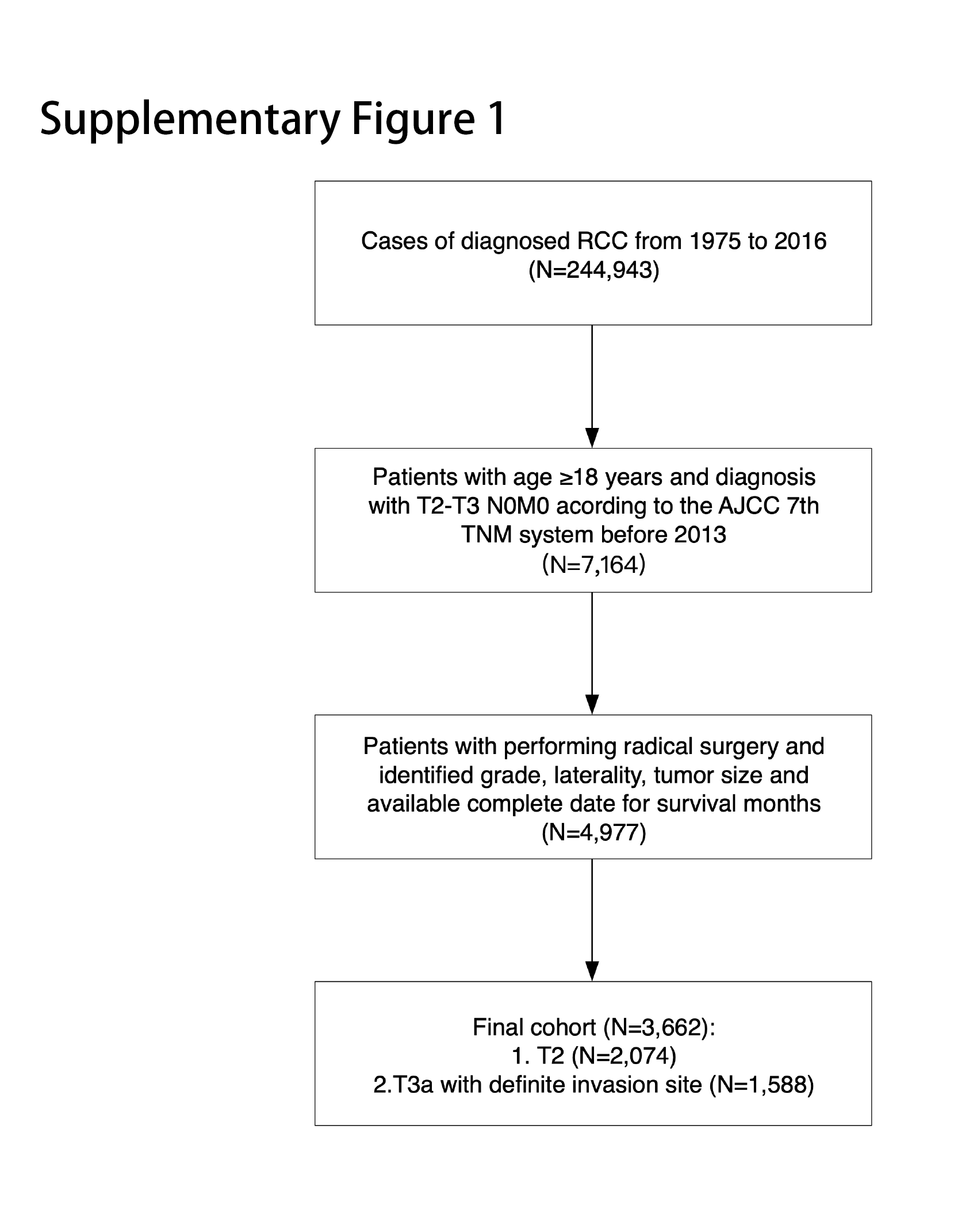

Supplement: Supplementary Figure 1 — Flow diagram for the patient selection from SEER. The encoding of the database is as follows. The renal cancer coded as primary site (C64.9), AYA site recode (8.5.1 carcinoma of kidney), and ICCC site recode ICD-O-3/WHO 2008 [IV (b) renal cancer]. Radical surgery coded as surgery of primary site (40 or 50). The invasion site of PFI coded as CS site-specific factor 1 (10) with CS extension (450 or 460). SFI coded as CS site-specific factor 1 (20) with CS extension (450 or 460). PFI+SFI coded as CS site-specific factor 1 (30) with CS extension (450 or 460). PFI+RVI coded as CS site-specific factor 1 (10) with CS extension (601). SFI+RVI coded as CS site-specific factor 1 (20) with CS extension (601). PFI+SFI+RVI coded as CS site-specific factor 1 (30) with CS extension (601). [file Image_1.TIF]

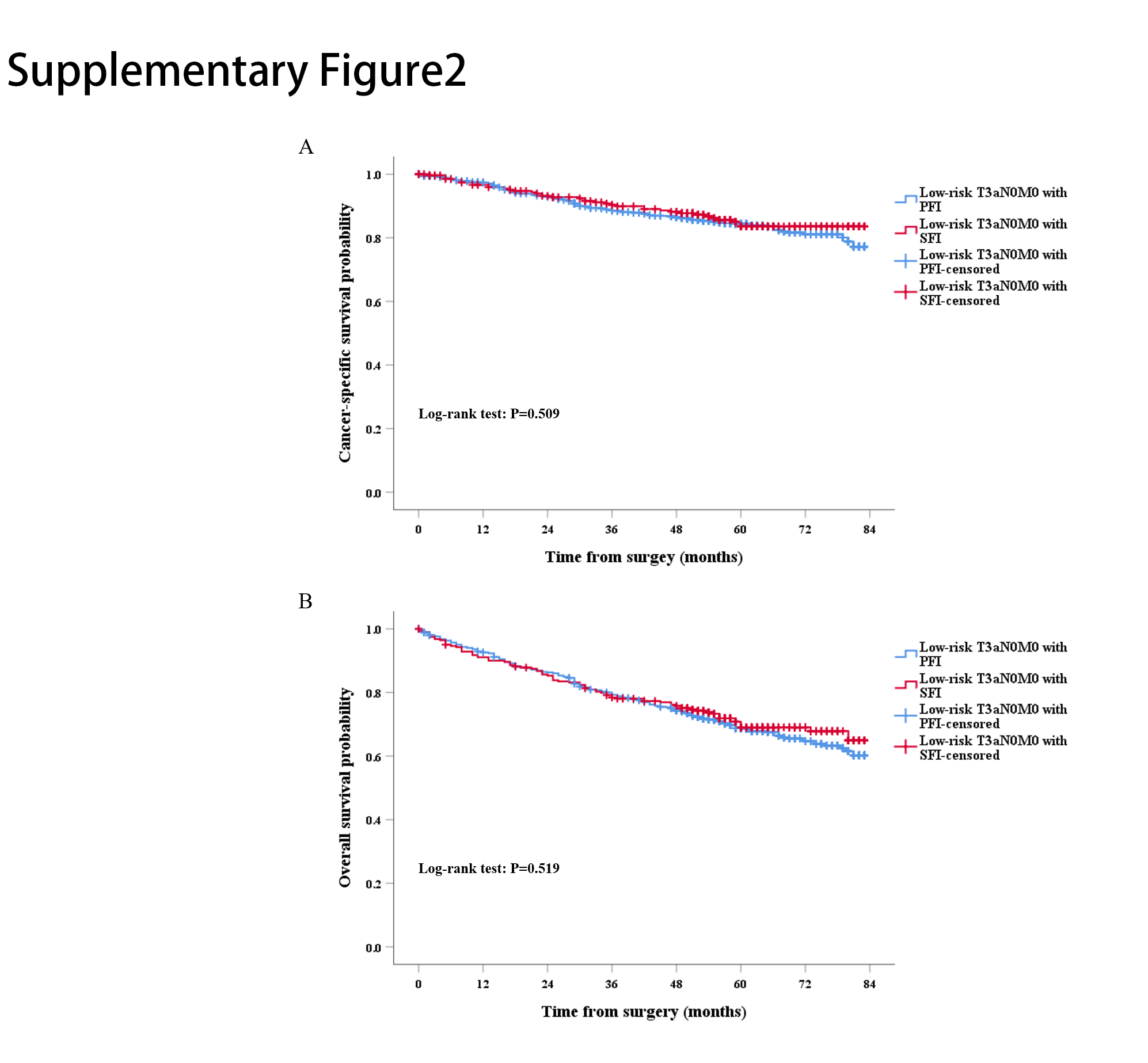

Supplement: Supplementary Figure 2 — Kaplan-Meier survival estimates for low-risk pT3aN0M0 RCC patients undergoing radical surgery with PFI only and SFI only for CSS (A) and OS (B). [file Image_2.TIF]

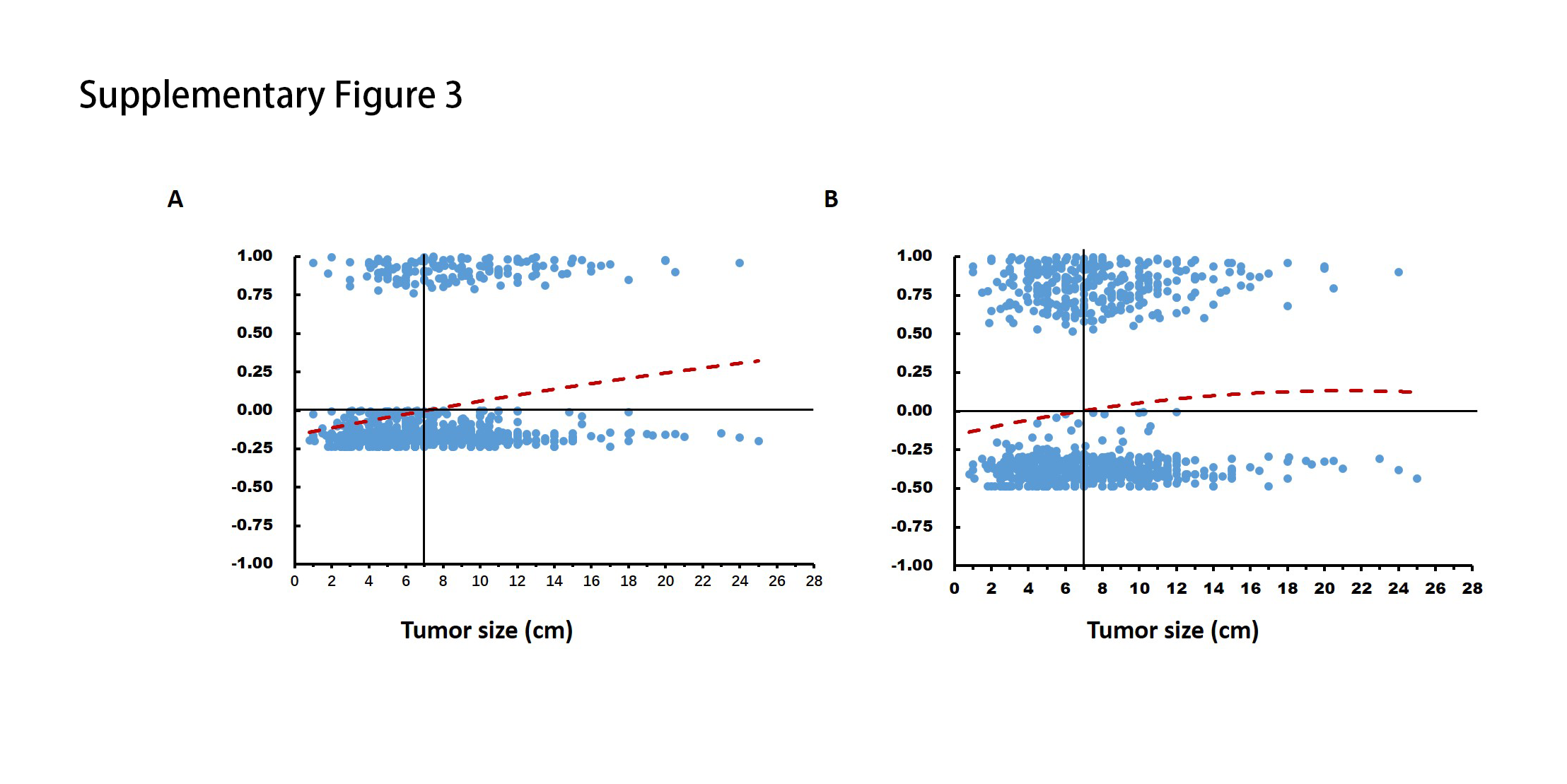

Supplement: Supplementary Figure 3 — Scatterplot of tumor size vs. Martingale residuals for 989 low-risk T3aN0M0 RCC patients undergoing radical surgery based on the CSS (A) and OS (B). The Martingale residual is the difference between the observed event and the expected numbers of events for a given patient, which indicates the expected risk of death for these patients. Compared with the expected risk based on Cox regression analysis, patients above the level line have a higher risk of death, while those below the level line have a lower risk of death. The figure shows that it is appropriate to distinguish these patients by 7 cm. [file Image_3.TIF]

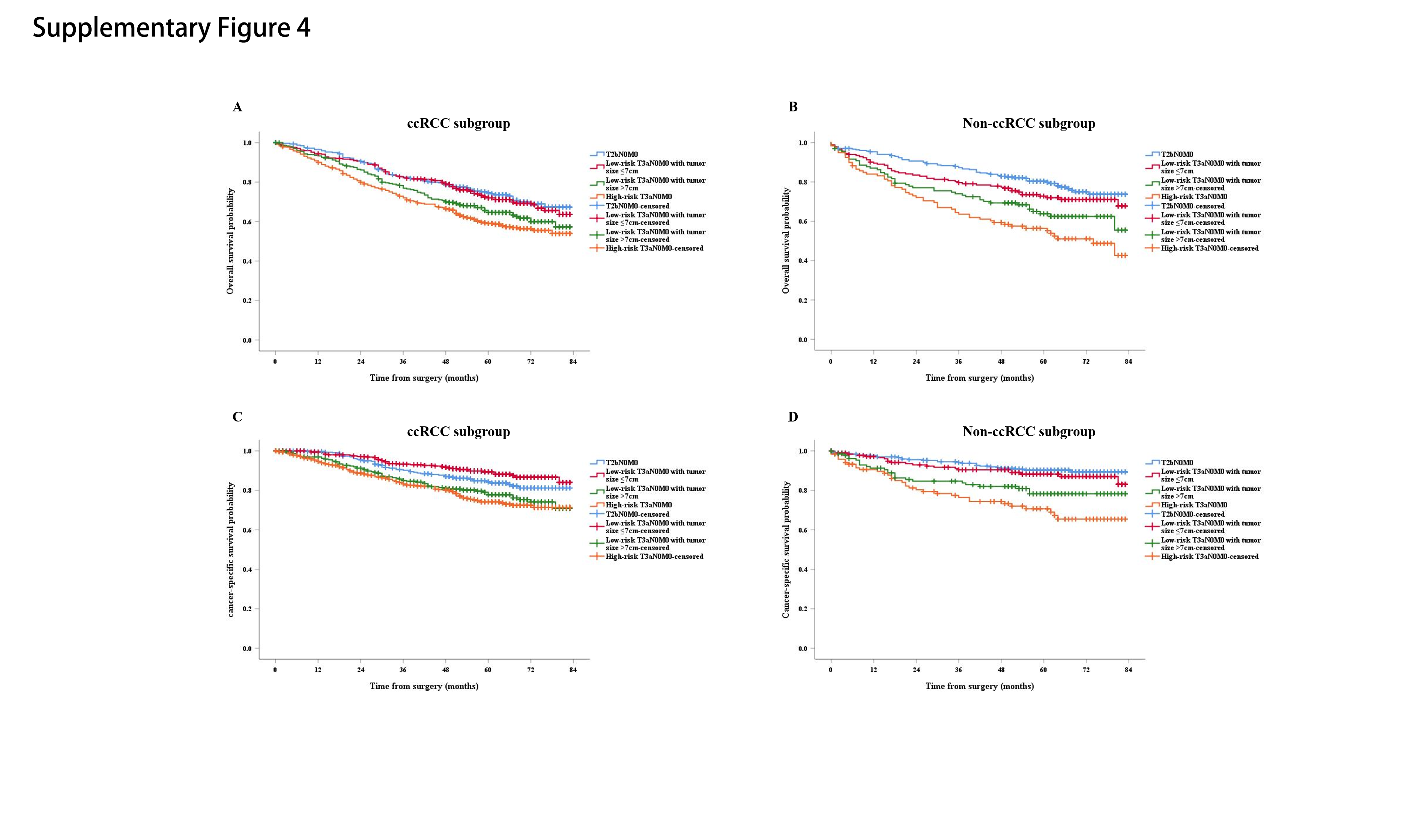

Supplement: Supplementary Figure 4 — Kaplan-Meier survival estimates for different staging patients undergoing radical surgery in the ccRCC and non-clear cell ccRCC subgroups for OS (A,B) and CSS (C,D). [file Image_4.TIF]
